# Research progress of brain organoids in the field of diabetes

**DOI:** 10.1186/s13041-024-01123-4

**Published:** 2024-08-06

**Authors:** Ying Su, Aimei Liu, Hongguang Chen, Qingjie Chen, Bo Zhao, Runze Gao, Kangwei Zhang, Tie Peng, Zhenwang Zhang, Changhan Ouyang, Dan Zhu

**Affiliations:** 1 Hubei Key Laboratory of Diabetes and Angiopathy, Xianning Medical College, Hubei University of Science and Technology, No.88, Xianning Avenue, Xianan District, Xianning, 437000 Hubei Province P. R. China; 2 School of Phamacy, Hubei University of Science and Technology, Xianning, 437000 Hubei Province P. R. China; 3 Hubei University of Science and Technology, Xianning, 437100 P. R. China

**Keywords:** Brain organoids, Diabetes, Pluripotent stem cells, Gene editing, Retinopathy

## Abstract

Human embryonic stem cells and human induced pluripotent stem cells may be used to create 3D tissues called brain organoids. They duplicate the physiological and pathological characteristics of human brain tissue more faithfully in terms of both structure and function, and they more precisely resemble the morphology and cellular structure of the human embryonic brain. This makes them valuable models for both drug screening and in vitro studies on the development of the human brain and associated disorders. The technical breakthroughs enabled by brain organoids have a significant impact on the research of different brain regions, brain development and sickness, the connections between the brain and other tissues and organs, and brain evolution. This article discusses the development of brain organoids, their use in diabetes research, and their progress.

## Introduction

Diabetes mellitus is a metabolic disorder that affects over 400 million people globally. It is characterized by hyperglycemia and may have a number of serious side effects, including early death [1].Beta-cell dysfunction is a consequence of type 1 diabetes (T1D), an autoimmune disorder. Type 2 diabetes (T2D), the most common form of adult diabetes, is characterized by peripheral insulin resistance and significantly incorrect insulin production [2]. Furthermore, rare monogenic diabetes mellitus is becoming more prevalent. These disorders, which include neonatal diabetes (ND) and mature-onset diabetes of the young (MODY), are caused by mutations in a single gene that is necessary for the development or function of pancreatic beta cells [2, 3]. Regretfully, there is still a great deal to learn about effective treatment options and the genesis of diabetes.

The term “organoids” refers to the technique of using stem cells cultured in vitro in a specific three-dimensional environment to create tissues with an architecture and functions similar to the original organ. The ability of stem cells to form complex tissue architectures and self-organize is necessary for the development of organoids. These self-organizing structures may include portions that reflect different brain areas; they are commonly referred to as “brain organoids” because of how frequently human brain regions are seen throughout the body. Cerebral organoids may have structural characteristics that are distinct to a particular region of the brain.

**Fig. 1:**
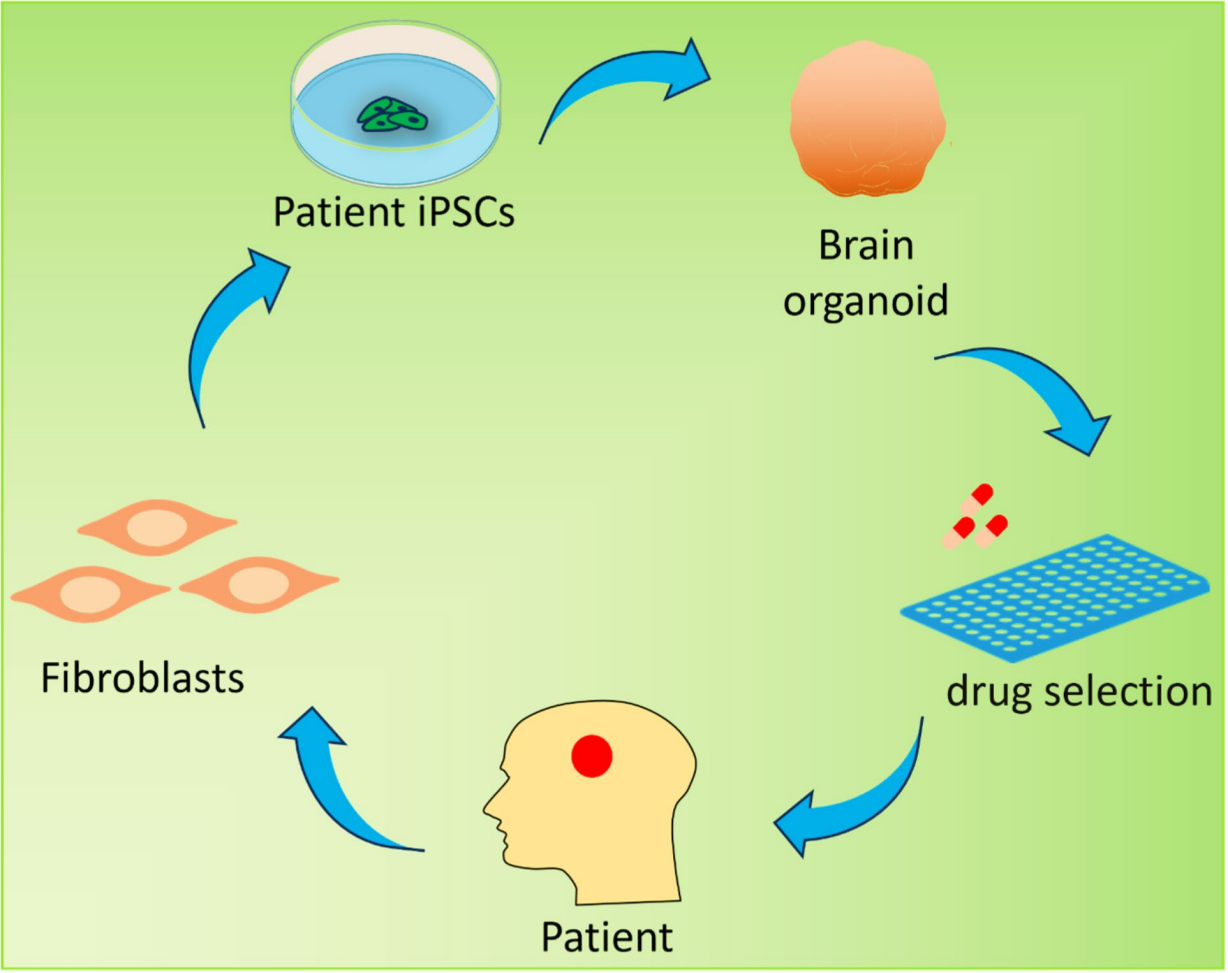
Brain organoid generation and therapeutic potential. Brain organoids can be generated from patient induced pluripotent stem cells (iPSCs) derived from adult fibroblasts and can be used to simulate human neurological disorders. Drug screening may be one of the potential applications for predicting drug efficacy before treatment

The novel technology known as organoids makes new models for the study of developmental biology and disease conceivable [4]. Human neurons are now widely used in in vitro systems that enable a wide range of phenotypic and mechanistic studies due to the recent and rapid advancements in stem cell technology, such as the ability to differentiate pluripotent stem cells (PSCs) and reprogramme somatic cells into induced pluripotent stem cells (iPSCs) [5, 6]. Modeling neuropsychiatric and neurological diseases using organoids derived from induced pluripotent stem cells (iPSCs) might be useful for drug discovery (Fig. 1). These efforts have recently led to the development of three-dimensional (3D) brain organoids, which are being used as experimental models to study the pathophysiology of disease and normal organogenesis [4, 7]. These organoids imitate the developing nervous system.

Brain organoids have significant promise for a variety of applications, including as drug screening, gene editing, modeling nervous system illnesses, exploring the evolutionary rules governing brain development, and modifying the brain’s evolutionary processes. There are several options for treating diabetes thanks to the recent and ongoing advancements in brain organoid technology. The composition, development, and use of brain organoids in diabetes are discussed in this article.

## Construction and development of brain organoids

### Research progress of brain organoid construction methods

Though not all 3D systems for neuronal culture could be regarded as brain organoids, methods to stimulate neuronal differentiation from RGCs into 3D neuronal structures have been pursued since the early 1990s. Brain organoids may replicate the brain’s developmental process and reflect the physiological, pathological, and pharmacological characteristics of the brain. They also have many anatomical and cellular similarities with the real brain [8, 9].

The creation of brain organoid technology is based on early research into the two-dimensional induction of neuroectodermal cells and the three-dimensional differentiation of embryoid bodies (EBs). Researchers also started looking at EB differentiation approaches because of the relatively basic cell types in two-dimensional (2D) culture systems, the stark disparities between cell interactions and actual tissue, and the challenge of directly examining human brain tissue. Advances in stem cell technology have made it possible for researchers to employ human induced pluripotent stem cells (hiPSCs) to construct brain-like tissues and organs from a 3D viewpoint. Researchers have also started to investigate neural cell differentiation procedures of PSCs [10, 11].

**Fig. 2:**
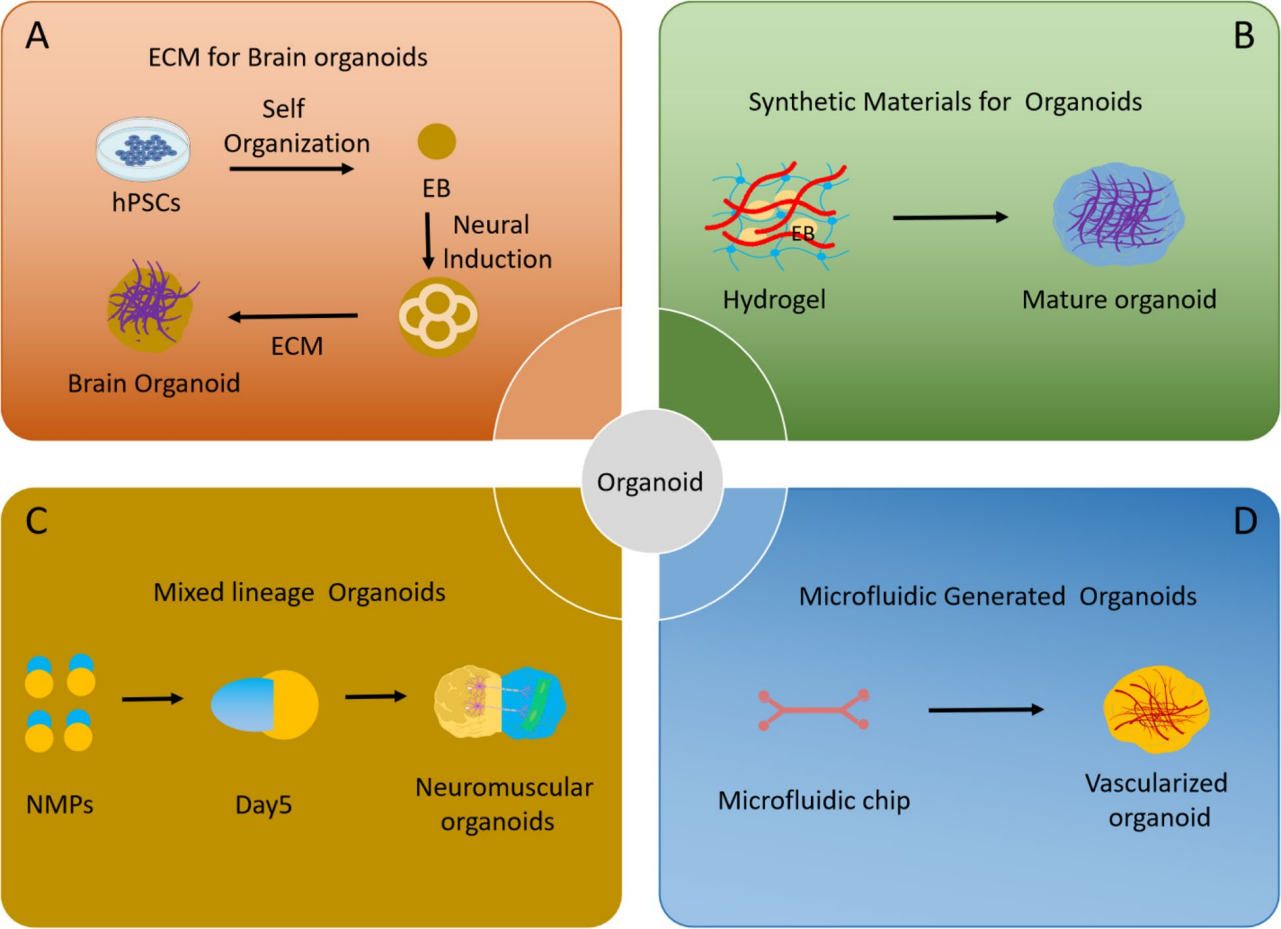
Advances in methodology of brain organoid generation. (**A**) A simple method using a minimum of medium and extracellular matrix to create self-organized brain organoids. (**B**) Synthetic materials promote organoid maturation. (**C**) Organoids with mixed systems, such as neuromuscular organoids, make it possible to study the interaction between organs. (**D**) Microfluidics develop vasculature in organoids

The creation of innovative procedures for brain organoid formation was made possible by the initial groundbreaking studies of differentiation employing 2D monolayer cultures [12–16] and the groundbreaking work on 3D cultures by the Sasai and Kleber group [17]. Generally speaking, there are two primary methods for creating brain organoids: VSC self-assembly and external sensor inputs. Using neural guiding molecules and extracellular arrays, the research groups of Sasai and Knoblich conducted experiments on 3D brain organoid culture systems [11, 13]. Key features of the fetal brain are mimicked by human brain organoids; nevertheless, the inability to create 3D brain structures that accurately represent late fetal development is due to the drawbacks of existing organoid techniques, including intrinsic hypoxia and cell death [18]. The development of a technique by Gordon et al. to create three-dimensional organoids of the human cerebral cortex with characteristics of the neonatal period is astounding [19] (Fig. 2).

Brain organoids have progressed from non-directional whole brain organoids to several brain organoids with distinct regional features, including cortex [20, 21], midbrain [22, 23], hippocampus [24], cerebellum [25], and spinal cord [26]. Specifically, realized brain organoid vascularity and regional brain organoids.

**Fig. 3:**
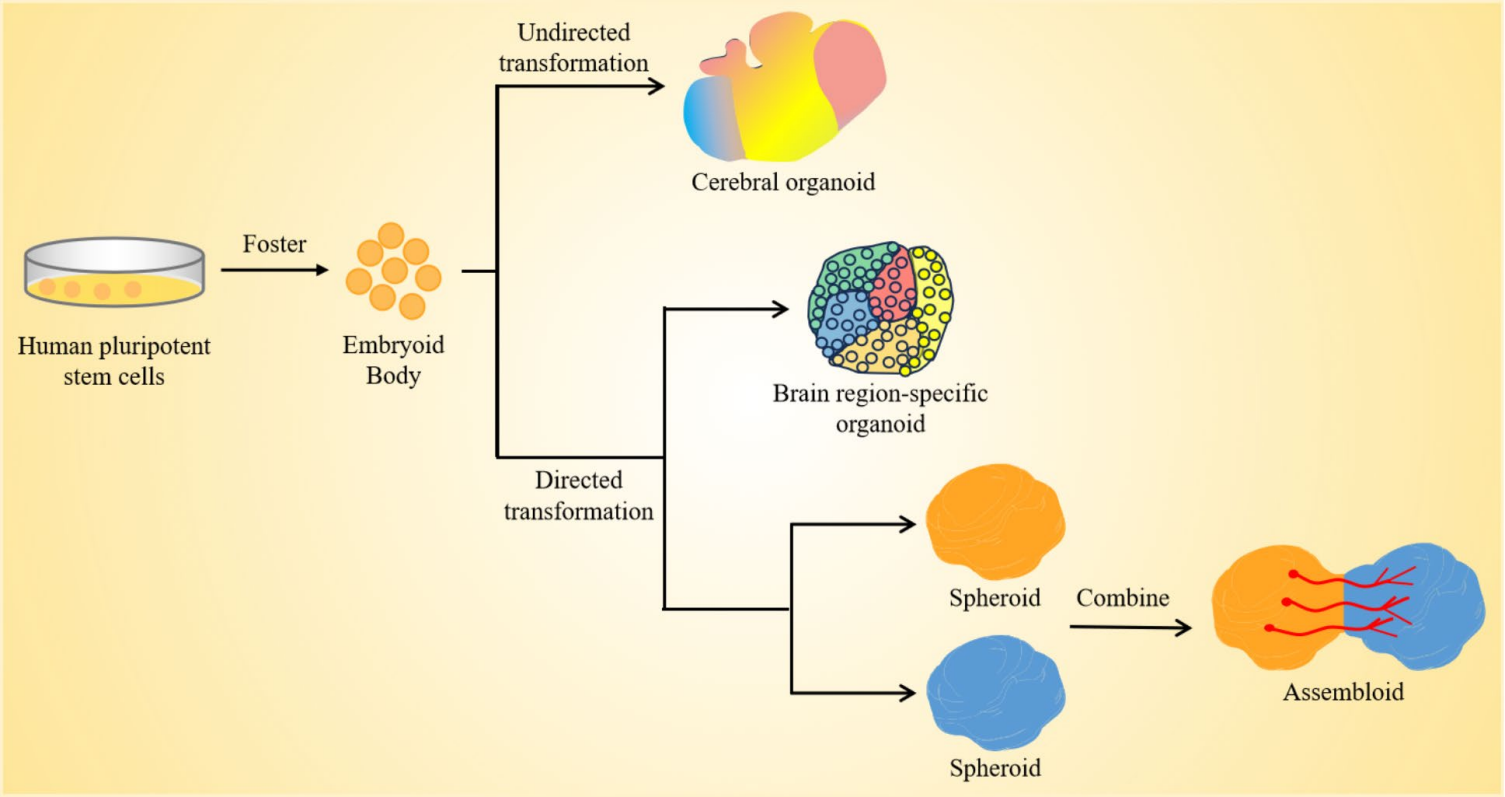
Unguided and guided approaches for making brain organoids. Unguided methods take use of hPSCs’ inherent signaling and self-organization abilities to allow them to naturally differentiate into tissues that resemble the growing brain. The resultant brain organoids often have diverse tissues that mimic various parts of the brain. Directed techniques use growth factors and tiny chemicals to create spheres that symbolize a particular tissue type. Organoid techniques specific to brain regions include the early usage of modular variables to influence the destiny of stem cells. Later phases of differentiation subsequently eliminate these components. Moreover, alignment techniques may be used to create two or more spheres or organoids that symbolize the identities of various brain areas. These can then be combined to create “class assemblies” that simulate the interactions of various brain regions

Currently, brain organoid cultures are classified into two categories based on whether or not targeted differentiation is carried out. First, stem cell differentiation produces organoid structures that, when grown in cultures, produce multi-brain organoids without the need for external morphogenetic agents. The second approach involves timing the addition of exogenous morphogenetic and neurotrophic substances to cultivate organoids in certain brain areas in accordance with the regulatory systems of the human brain development process (Fig. 3).

### The non-oriented brain organoids

Serum-free culture was primarily used to create neuroectoderm during the early stages of brain organoid differentiation [27], while matrigel and bioreactors were used to accomplish long-term neuronal differentiation culture [11], among other methods. The SFEBq culture method, for example, successfully differentiates embryoid bodies of mouse embryonic stem cells (mESCs) into telencephalic tissue by simultaneously adding inducers for neuronal differentiation (e.g., Wnt antagonists and Nodal antagonists). Sasai’s team [28] produced a serum-free liquid culture of embryoid body-like aggregates with rapid reaggregation in 2005. By blocking the Notch pathway, the team used SFEBq to create human and mouse embryonic stem cells (ESCs) that resemble the retina in terms of composition and cell structure, so mimicking the retina’s developing process to some degree. He established the foundation for the creation of brain organoids [18, 29].

Using the SFEBq technique, Lancaster et al. used hiPSCs for the first time in 2013 to promote differentiation into entire brain organoids [11] (Fig. 2). They stimulated and directed the differentiation of hiPSC embryoid structures with endoderm, mesoderm, and ectoderm. After that, they went through neuroectoderm and neuroepithelium to produce structures that resembled the early embryo’s cerebral cortex, which may have represented the early embryo’s development of the human brain.

Cells linked to the hippocampus, retina, forebrain, midbrain, hindbrain, and other areas with apico-basal polarity are seen in whole-brain organoids [11]. Additional research has shown that the integration of tissue engineering and three-dimensional culture may enhance the tissue structure of brain organoids and boost the repeatability of differentiation [30]. Researchers used microfilaments of poly (lactide-co-glycolic acid) (PLGA) fibers as scaffolding to generate microfilament-engineered brain organoids (MEOs) in 2017 [31]. The brain organoid scaffolds made from microfilms are part of a system that advances cortical development and encourages the production of neuronal ectoderms [31]. He used air-liquid interface culture of whole brain organoids (ALI-COR) in 2019; this method improves axonal development and neuron survival in whole brain organoids [32].

To put it briefly, the unguided brain organoids created in this work mimic the cellular makeup and anatomical structure of the brain in vivo, can model the brain’s developmental process, and can represent the physiological and pathological characteristics of the brain. These capabilities open up new avenues for research on brain function, disease simulation, drug discovery, and other related topics.

### Region-specific brain organoids

Organoids made out of the whole brain exhibit unique heterogeneity [33]. Brain organoids were developed to mimic specific functional properties of individual brain regions in order to better understand the functions of various brain regions and their interregulation, as well as to look into patterns of neuronal development and the onset and progression of diseases in particular brain regions. Organoids of the brain were created to mimic the properties of several brain areas, including the cerebellum, midbrain, and forebrain (Table 1).

**Table 1:** Brain organoids derived from pluripotent stem cells

Organoid type	Unguided/guided	Brain region	Cell type used	Culture conditions	References	
Cerebral organoid	Unguided	Whole brain	Mouse ESCs, hESCs, human iPSCs	Suspended, rotating bioreactor	[11]	
	Unguided	Forebrain	Human ESC	Microfibers, suspended, rotating horizontal shaker	[31]	
	Unguided	Forebrain	Human iPSC	Microfibers, suspension, rotating horizontal shaker, air-liquid interface	[32]	
Serum-free culture of EB-like aggregates (SFEBq)	Guided	Cerebral cortex	Mouse ESCs, Human ESCs	Stationary floating culture, followed by replating of aggregates	[28]	
Forebrain organoid	Guided	Cerebral cortex	Human iPSCs, Human ESCs	Miniaturized multi-well spinning bioreactor	[34, 82]	
Optic cup organoids	Guided	Optic cup	Mouse ESCs	Floating culture in 40% oxygen /5% carbon dioxide	[18]	
Thalamus organoids	Guided	Thalamus	Human ESCs	Floating culture, rotating horizontal shaking table	[35]	
Hypothalamus organoids	Guided	Hypothalamus	Human iPSCs	Floating culture, rotating bioreactor	[34]	
Cerebellar organoids	Guided	Posterior brain /cerebellum	Human ESCs	Floating culture	[26]	
Midbrain organoids	Guided	Midbrain	Human ESCs	Floating culture, rotating horizontal shaking table	[24]	
Cortical organoids	Guided	Cortex	Human ESCs	Floating culture in 40% oxygen/5% carbon dioxide	[18, 30]	
Pituitary organoid	Guided	Anterior pituitary	Human ESCs	Stationary floating culture	[83]	
MGE organoids	Guided	medial ganglionic eminence	Human ESCs	Suspended, rotating horizontal shaker	[41]	
Hippocampal organoids	Guided	Hippocampus	Human ESCs	Floating culture in 40% oxygen/5% carbon dioxide	[25]	
Fused cerebral organoids	Guided	MGE and cortex	Human ESCs	Suspended, rotating horizontal shaker	[41]	
	Guided	Ventral and dorsal forebrain	Human ESCs	Suspended, rotating horizontal shaker	[39, 40]	
	Guided	Thalamus and cortex	Human ESCs	Suspended, rotating horizontal shaker	[35]	
	Guided	Cortex and striatum	Human ESCs	Suspended	[42]	

The optic nerve, hippocampus, thalamus, hypothalamus, and other brain areas are included in the forebrain. In 2011, Sasai et al. caused PSCs to spontaneously generate upper hemisphere vesicles [18], inhibiting the WNT pathway and concurrently activating the WNT pathway to establish a proximal-distal axis. The distal portion folds inward to create the optic cup structure, while the proximal section includes retinal features including inner nuclear layer (INL) and retinal ganglion cells (RGCs). The research produced organoids called optic cups that contained several kinds of retinal cells. After that, the scientists constructed a 3D culture system that resembled cortical growth [21, 22], setting the stage for the production of organoids of the brain that are particular to a certain area. 2015 Sasai and colleagues optimized cortical organoids and produced hippocampal organoids by inhibiting SMAD pathways and activating WNT and BMP pathways [25]. In 2016, Qian et al. created a hypothalamus organoid by inhibiting the SMAD route and activating the WNT and SHH signaling pathways [34]. In order to create neuroectodermal organization and increase BMP7, Xiang et al. blocked the SMAD pathway in 2019 [35]. This resulted in the development of thalamic organoids.

In the pathophysiology and therapy of Parkinson’s disease (PD), the midbrain—which regulates information transmission, motor control, and sensory processing between the forebrain and spinal cord—has drawn a lot of interest. Jo et al. produced midbrain organoids in 2016 by adding SHH/FGF8 to induce ceiling structure [24], activating the WNT pathway, and inhibiting the SMADs pathway. By identifying dopamine production, they opened up a new avenue for research on Parkinson’s and other illnesses. Furthermore, in 2015, MONZEL et al. stimulated neuroepithelial stem cells to produce organoids in the midbrain using SHH inhibitors and GSK3 inhibitors [23].

The brain’s motor control center, the cerebellum, has the capacity to develop into distinct neuronal groupings. To form cerebellar organoids, Muguruma et al. created a boundary structure between the midbrain and cerebellum by blocking the SMADs pathway, adding FGF2 and insulin to promote caudation of cerebellar organoids, and then adding FGF19 and SDF1 to induce cells to promote neuroepithelial formation in the cerebellar lamina [26]. Because of the diverse cell types and fragile structure of the cerebellum area, the cerebellar organoid culture system still needs a long-term culture system.

In addition to neurons, microgila also play a pivotal role in the brain’s functionality. Currently, the lack of microglia with the ability to reshape neuronal networks and phagocytose apoptotic cells and debris is a major shortcoming of the midbrain organoid system. Moreover, modeling of diabetes-related neurological complications is not possible in the absence of microglia. By co-culturating hiPSCs-derived mesodermal progenitor cells (Brachyury^+^) with neurospheres, Worsdorfer et al. renewably generated vascularized neuroorganoids that included vasculoid structures (CD31^+^) and microglia-like cells [36]. This study provided a model for studying angiogenesis and neurodevelopment, but did not investigate the function of microglia in the organoids. In the another study, Fagerlund et al. reported that hiPSCs-derived eythro-myeloid progenitors (CD41^+^) migrated into human brain organoids [37]. Differentiated into microglia-like cells, and interacted with synaptic material. Whole-cell patch-clamp and multi-electrode array recording showed that microglia within organoids promoted the maturation of neural networks. A recent study that co-cultured human midbrain organoids with hiPSCs-derived macrophage progenitor cells also reported that microglia integration let to increased nerve maturity and function [38]. Whole-cell patch-clamp and multi-electrode array recordings showed that lower action potential generation thresholds and shorter peak-to-peak intervals were observed in midbrain organoids with microglia integration, suggesting that microglia integration improve neural maturation.

The methodologies for constructing region-specific brain organoids are also examined. The development of these organoids continues to advance, integrating developmental inducers, biomaterials, and bioreactor systems. It is anticipated that more precise realization of region-specific brain organoids can be achieved. The aim is to replicate the maturation processes of various brain regions and establish relevant disease models. These may be used to research the control of neurodevelopment and the beginning of illness in certain brain areas. To more accurately mimic the physiological structure of the brain, however, further work has to be done on the precision of the generated brain areas and the repeatability of the cells.

### Brain organoid fusion

Although brain organoids may be utilized to model many interacting brain areas, their sizes and spatial configurations are very varied and unpredictable. Researchers have attempted to mimic the structure and environment of the genuine human brain by integrating several brain organoid areas in an effort to create a more realistic brain organoid design that can replicate the development of different brain regions and model disorders. 2017, Team Pasca performed neural induction of PSC by SFEBq to induce the formation of dorsal and ventral telencephalic organoids by regulating WNT and SHH signalling [39], spontaneously fused ventral and dorsal telencephalic organoids and observed irregular migration of interneurons in the cortical tissue. Interneurons in fused organoids from Timothy syndrome patients migrated abnormally. Bagley et al. fused the ventral telencephalon and whole brain organoids to form brain organoids fused to the dorsoventral axis [40]. Based on this, Xiang et al. studied the migration of CXC chemokine receptor 4 (CXCR4) dependent interneurons from ventral to dorsal migration [41]. Medial ganglionic neurite (MGE) organoids were constructed, and then fused with cortical organoids to examine CXC chemokine receptor (CXCR4) dependent interneurons.

To better imitate the mutual projection of the thalamus and cortex, XIANG et al. produced thalamocortical fusion organoids by physically combining thalamic and cortical organoids [35]. Studies of neurological conditions including schizophrenia and autism spectrum disorders may be conducted using the biaxial projection between the thalamus and cortex, which replicates synaptic connections in the body. Subsequently, MIURA et al. employing fusion of striatal and cortical organoids, showed that cortical neurons project axons to striatal organoids and make synaptic connections with neutral invertebrate neurons, showing enhanced electrical features and calcium activity [42]. By combining the two kinds of organoids, functional integration was accomplished in these four investigations. In an in vitro three-dimensional culture media, they replicated the tangential movement of human endoneurons [39–42].

Brain organoid fusion methods provide a potent platform for investigating the relationships between various brain regions/tissues, including the impact of tissue growth centers on brain organoid development and the investigation of cell-cell interactions in vitro. Nevertheless, in order to create functioning circuits and provide helpful instruments for researching brain function, current fusion organoid manufacturing techniques need to be further refined to represent particular brain space projections and physiological reactions.

### Vascularization of brain organoids

Organoids in the brain still differ significantly from the genuine human brain. Lack of a circulatory system is one of the main obstacles. Gas penetration, nutrition delivery, neuron differentiation, and other processes are all impacted by vascular function [43, 44]. Necrotic regions will form in the organoid center as a result of inadequate oxygen and nutrient penetration, which will interfere with the proper growth of brain organoids and the neuronal migration route [45]. Thus, the development of a vascular network is a critical requirement for the optimization of brain organoids. As of right now, there are primarily two methods for vascularizing brain organoids: creating blood vessels in the organoids by in vivo transplantation and creating blood vessels in vitro.

In 2018, researchers transplanted brain organoids into the cerebral cortex of NOD-SCID immunodeficient mice, and the blood vessels of mice infiltrated into the implanted brain organoids within 14 days after transplantation; Compared with non-vascularized brain organoids in vitro, the in vivo development environment improves cell maturation and survival in brain organoids [46]. For the purpose of achieving the functional link between human axons and neurons in the mouse brain, brain organoids that have been transplanted may produce a lot of new neurons and live for over 200 days [46]. To create human-mouse vascular tissue linkages in the grafts, human vascularized organoids (vOrganoids) co-cultured with human umbilical vein endothelial cells were inserted into the mouse S1 cortex. Compared to non-vascularized brain organoids, vascularized brain organoid transplantation increases blood vessel development and cell survival [47]. As a result, a series of transplantation experiments have demonstrated the importance of vascularization in the maturation of brain organoids.

In terms of in vitro vascularization, in 2019, Cakir et al. co-differentiated human embryonic stem cells expressing ETV2 (ETS variant 2) with wild-type embryonic stem cells to achieve directional induction of vascular endothelial cell differentiation in cortical organoids, based on which vascularization cortical organoids were constructed [48]. vascularized human cortical organoids (vhCOs) form perfusable blood vessels; Compared with control cortical organoids, the cell survival rate in vascularized cortical organoids was significantly improved [48]. In addition, by co-culturing with venous endothelial cells, researchers were able to establish vascularized brain organoids in vitro, in which venous endothelial cells can form well-developed reticular or tubular vascular systems, as confirmed by single-cell RNA sequencing. This vascularized brain organoid system has similar molecular properties and cell types to the human fetal telencephalon [47]. The integration of brain organoids and vascular system under in vitro culture will help to improve the phenomenon of central necrosis during the long-term cultivation of brain organoids.

## Application of brain organoid research technology in the field of diabetes

**Fig. 4:**
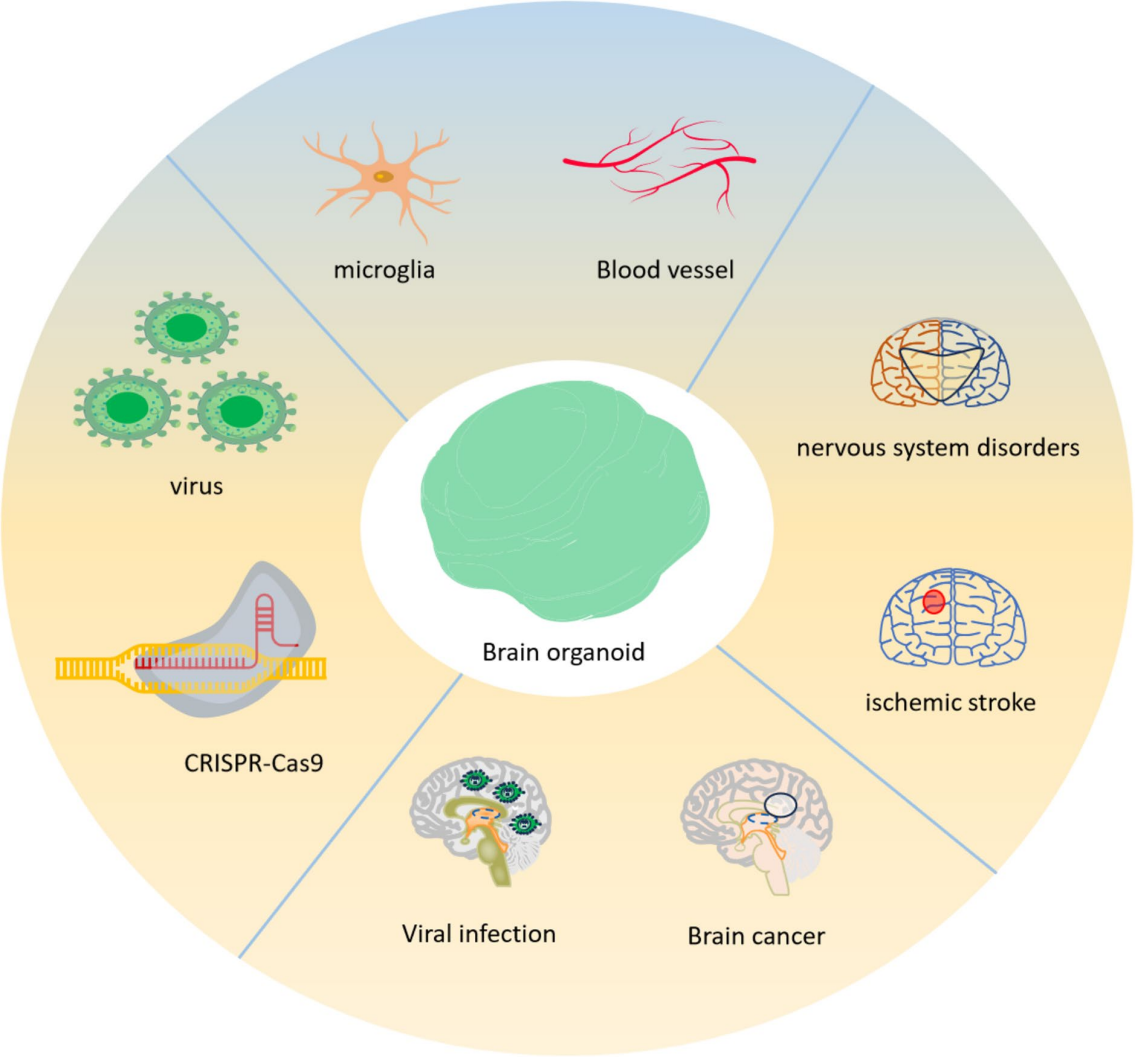
Applications of brain organoid research. As part of a regenerative medicine therapy, pluripotent stem cells (PSCs) from brain organoids may be implanted to brain injury areas to heal damaged tissue or utilized to investigate brain illnesses. To simulate vascular and infectious disorders and investigate their interactions with organoid cells, non-CNS derived entities including microglia, blood arteries, and viruses may be incorporated into brain organoids. The genesis of disorders affecting the nervous system may be studied using brain organoids obtained from patients or genetically engineered using CRISPR-Cas9 to carry disease-associated genetic abnormalities

As a novel in vitro cultivation technology, brain organoids may not only mimic early brain development in vitro, but also help to understanding brain development and developmental paths. In addition, brain organoids provide novel tools for neurological illness modeling, in vitro drug screening, gene therapy, and the simulation of human brain development (Fig. 4).

The uses of brain organoids for illness [49], drug development [50], evolution [51], and brain development [40] may be further expanded by combining gene editing methods with brain organoids to generate various sorts of mutations. Brain organoids may also be used in conjunction with single-cell sequencing technology, which is crucial for understanding how the brain develops and figuring out how diseases are caused. Recent years have seen significant developments in the field of diabetes, offering fresh perspectives on the management and prevention of diabetes-related disorders, thanks to the quick growth of brain organoid research technologies.

### An organoid brain model associated with KCNJ11 p.V59M was used to examine the pathogenic mechanism of neonatal diabetes

The brain organoid approach is a good model for studying neuronal differentiation and the consequences of genetic variety on brain development and disease gene expression [52–54]. Neonatal diabetes (NDM) has been studied through the direct effects of P. Vir59met (V59M) in the KCNJ11 gene on precursor neurons and neuronal cells. Gokhan et al. generated brain organoids from martyrs or hiPSCs with the KCNJ11 V59M mutant allele to isolate confounding effects associated with NDM [55].

The pancreas and the brain express the KCNJ11 gene, which genes for Kir6.2, a crucial subunit of the ATP-sensitive potassium channel (KATP). NDM may result from the acquisition of a functional mutation in heterozygosity in the KCNJ11 gene. A dominant heterozygous mutation in the KCNJ111 gene causes NDM, a monogenic illness that affects around 30% of the population. These mutations often cause the KATP channel to be permanently activated, which keeps the cell persistently hyperpolarized. Due to the altered functionality of these KATP channels, the beta cells within the islets are incapable of secreting insulin, Consequently, this results in elevated blood sugar levels [56]. Neural stem cells in V59M organisms often do not develop and move, according to data. As a result, there are abnormalities in the development and function of brain circuits. This lowers neurogenesis. Tolbutamide (Tol), a KATP channel blocker, may be used as a medication to treat mutant organoids, which can partly correct the molecular flaws brought on by the cell membrane’s hyperpolarization. In brain tissue taken from HIPSC patients, this work offers the first concrete proof that the mutant KCNJ11 channel results in neurological impairment.

New drug therapies for people with inherited diseases can typically be found thanks to advancements in personalised medicine platforms that use stem cell-derived tissue [57, 58]. Additionally, pathology linked to mutations in the KCNJ11 gene for neonatal diabetes can be detected thanks to hiPSC-derived brain organoid platforms. Using a brain organoid platform, confounding effects associated with neonatal diabetes were differentiated from the direct impact of V59M mutations on neurocytes and neurons. It may be inferred from this that the development of brain organoids offers valuable insights into the fundamental principles of cellular and neurophysiological events linked to intricate metabolic disorders.

### Treatment of diabetic retinopathy with cerebral organoids containing optic vesicles

In recent years, various 3D brain organoids including hiPSC-derived neuroretinal discs have presented new chances to research retinal illnesses [39, 59, 60].

**Fig. 5:**
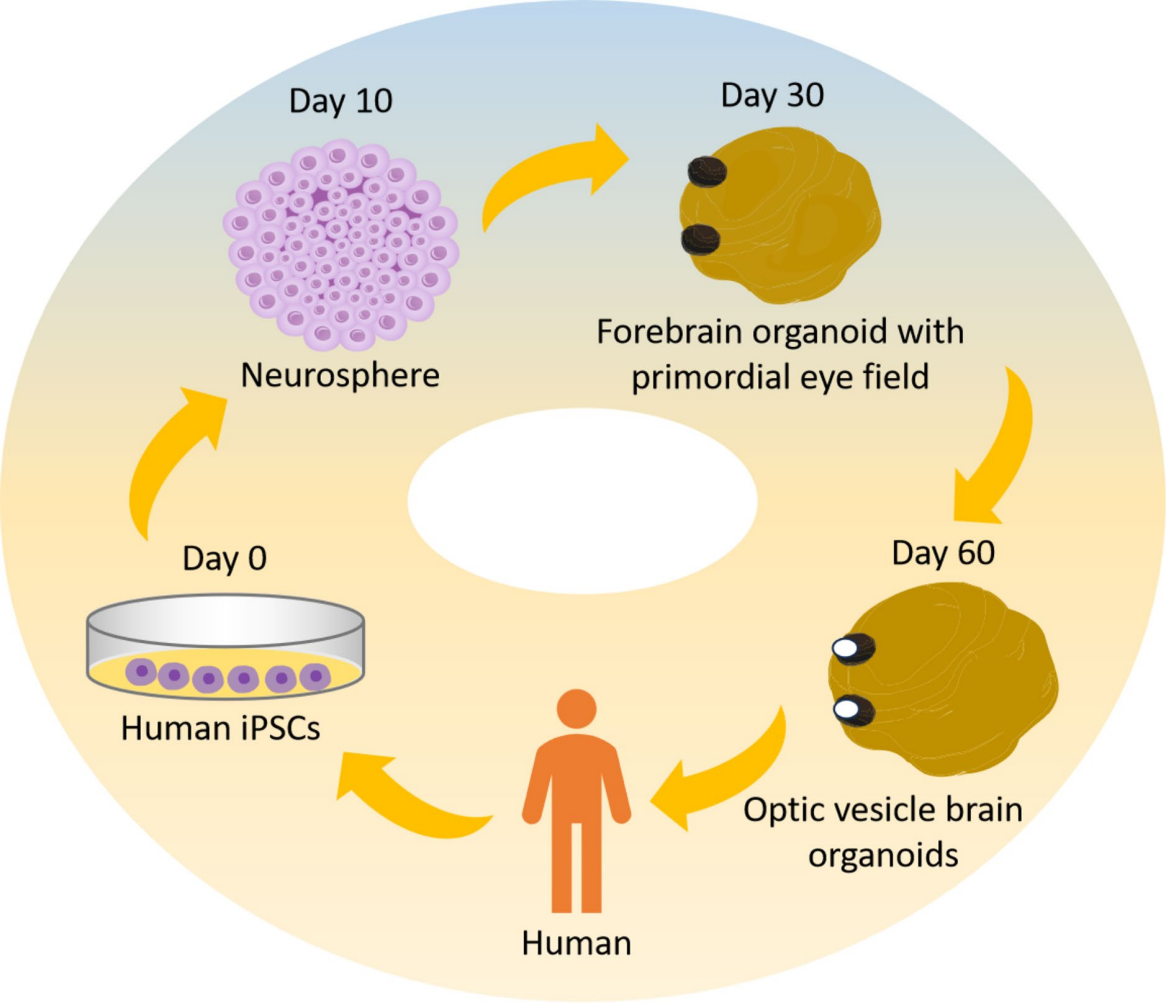
Schematic showing steps of OVB-organoid generation from iPSCs. The results of the research demonstrated the use of IPSC-derived human brain organoids in the generation of bilateral forebrain-connected OV, cellular diversity, and complexity reduction. The brain organoids start to gather OV at day 30, and during the next 60 days, they manifest as distinct structures

By altering the growth conditions to transform iPSCs into neural tissue, Elke et al. were able to successfully create bilaterally symmetric visual slices in brain organoids in 2021 [61]. The researchers began the induction culture with a decreased cell density. After that, during the neuroectodermal growth phase, retinol acetate (RA) was given to the media at various concentrations. Staining structures, most likely the original “eyes,” emerged in the tissue cultivated with retinol acetate after about 30 days in culture (Fig. 5). Immunofluorescence labeling indicated considerable expression of the eye-associated marker genes RAX, Pax6 and FOXG1 in the stained areas of these organoids.

Subsequent examination revealed that FOXG1 expression was gradient in the SOX2-positive invagination zone, suggesting that the visual and forebrain regions in these organoids were distinct, in line with the eye’s maturation process throughout human embryonic development. After a growth period of 50–60 days, the rudimentary “eyes” transformed into one or two mature, visible optic nerve cell structures (Fig. 5), and such organoids are designated optic vesicle cell-brain organoids (OVB organoids). This work is the first to functionally connect brain organoids with retinal structure. It helps to explore the interplay between “brain and eye” during embryonic development and offers a strong tool for the pathophysiology and treatment of diabetic retinal disorders.

Based on this, scientists have cultivated exosomes from tissues resembling OVB, which are involved in both retinal development and retinal disorders including diabetic retinopathy [62].

The primary symptom of diabetic microangiopathy, which causes distinct alterations in the retinal lesions, is diabetic retinopathy. Furthermore, retinal ischemia-reperfusion damage (IRI) is a significant factor in the development of DR [62]. Directly after retinal ischemia are transient hypoxia and nutritional depletion. Reperfusion produces excessive reactive oxygen species (ROS), which leads to oxidative stress and an exaggerated inflammatory response [63]. The significance of mesenchymal stem cells (MSCs) in vitro exosome secretion in the eye has gained more interest since studies have shown that intravitreal injection of LV-derived exosomes may alleviate diabetic retinopathy (DR) [64]. Moissiev et al. established the impact of exosomes on retinal ischemia by intravitreally injecting hypoxia-grown exosomes containing angiogenic active components and control saline into mice models of oxygen-induced retinopathy (OIR) [65]. Exosome therapy decreased and avoided retinal thinning in comparison to the control group.

Moreover, Liu et al. discovered that exosomes may transport endogenous miR-579 and circulating RNA cPWWP2A, and that these effects on retinal vascular function in diabetes patients are mediated by differences in the expression levels of these two molecules [66]. 148 Adipose tissue mesenchymal stem cell exosomes carrying miR-192 or miR-222 have been shown by Safwat et al. to be able to suppress angiogenesis and inflammatory responses in the DR [67].

The potential to cure diabetic retinopathy has increased with the discovery of OVB organoids. OVB organoids are a significant tool for disease modeling, high-throughput drug screening as an alternative to animal models. An affordable substitute for animal models in illness research and medication screening are in vitro models. Exosomes produced from brain organoids seem to offer tremendous promise for medication delivery and the treatment of ocular illnesses, according to recent research on the therapeutic effects of exosomes in ocular diseases. Furthermore, a great deal of obstacles still need to be addressed, such as the absence of microglia and blood arteries, which are essential for preserving the long-term survival of organoids and accurately replicating the retina. In addition, novel biotechnologies including 3D bioprinting, oxygen delivery systems, and retina-on-a-chip are being created to meet these difficulties.

### Building novel and emerging organoid culture systems to simulate organoid co-culture microenviroment in diabetes disease modeling

The properties and uses of brain organoid systems can currently be further increased when paired with additional engineering methods. Organ-on-a-chip technology offers a critical platform for the facile manipulation of the microenvironment and nutrient supply, and it constitutes a pivotal method for the concurrent culture of diverse cell and tissue types within organoid systems [68]. Furthermore, organ chips mitigate the challenge of co-culturing distinct organs of the same type in a singular medium to a considerable extent. The ability to replicate the interplay of several organs in an in vitro setting is extremely important, particularly for complicated metabolic illnesses that impact multiple tissues, like diabetes mellitus. A microfluidic in vitro model is employed for simulating neural tube development. For instance, an organ-on-a-chip configuration may utilize soluble factor-infused microchannels as entry and convergence zones, facilitating the establishment of a consistent morphogenetic gradient via diffusive processes within a central culture chamber. This system was able to replicate the Sonic Hedgehog (SHH) signaling gradients and the bone morphogenetic protein (BMP) gradients along the dorsoventral axis of the neural tube, thereby facilitating the induction of neural tube development models [69].

In 3D cultured organoids, material diffusion and transport are not enough to meet the growing metabolic demand, so it is difficult to ensure long-term growth and maturity. The establishment of functional vascular system is a necessary condition for the continuous healthy cultivation of brain organoids. Capabilities of perfusable blood vessels can be emulated through the utilization of organ-on-a-chip technology [70–72]. In addition, microchips or micro-bioreactors have also been constructed for brain organoids, such as in microfluidic systems that reduce necrotic areas in midbrain organoids by ensuring the supply of media through continuous laminar flow [73], which utilizes forced convection and media mixing to enhance nutrient supply. Therefore, organ chips is also an important solution to solve the difficulties faced in the process of organoid culture [68].

## Summary and prospect

Recent developments in the field of organoid technology have yielded substantial improvements in our comprehension of the principles behind human brain development and the etiology of neurological disorders. By combining various technologies, scientists have made significant progress in understanding the evolutionary laws of brain development and the mechanisms that regulate brain development. These models, which include non-targeted, regional, and combined brain organoids, hold promise for modeling nervous system diseases, drug testing, gene editing, and other areas.

Nevertheless, the present technology for producing brain organoids has not been created yet and has significant limitations because to the constraints of culture and induction approaches. The size of organoids, the maturation of neurons, and the subsequent generation of more complete cell types are all limited by the conditions of in vitro culture. Additionally, because brain organoids lack complete neuronal circuits and functional zoning, it is difficult to predict internal structures such as oligodendrocytes and astrocytes. Finally, the application of brain organoids is limited because they lack essential cell types like immune cells, which cannot form complex neuronal circuits. Thirdly, brain organoids’ metabolic properties vary greatly from those of the real brain. Fourthly, the growing conditions of brain organoids and the chemical combinations introduced vary due to the heterogeneity of iPSC cells in various labs, resulting in wildly disparate brain organoid models. Fifth, the cell composition, morphological features, and differentiation efficiency of the various batches of brain organoids vary as well [8].

The use of brain organoids in diabetes therapy is still in its infancy. In 2021, Gokhan et al. separated the direct effects of the V59M mutation on neuroprecursors and neurons from the disruptive effects linked to neonatal diabetes using the brain organoid platform [55]. Electrophysiological investigations have revealed that mutant brain organoids may create functioning neural networks whose excitability is reduced under baseline circumstances, even if mutant KCNJ11 channel activity hinders the formation of neuronal precursor cells. Furthermore, raising extracellular potassium levels erased the difference between the amount of mutant brain organoids and spontaneous active control. According to the findings, in human samples, mutant KCNJ11 channel activity does not control network excitability or circuit development. The method by which brain organoids may more effectively regulate the cellular and neurophysiological processes that accompany complicated metabolic disorders is supported by the findings of this research. This is a novel approach to the treatment of diabetic neuropathy using brain organoids in research.

Furthermore, new therapeutic approaches for diabetic retinopathy have been made possible by the discovery of OVB organoids as well as complicated brain and retinal multiorganoids. CPWWWP2A cyclin RNA was found in exosomes produced from OVB organoids by Liu et al. [66]. This finding may indirectly affect retinal vascular function in diabetes patients. Exosomes rich in miR-192 or miR-222 produced from mesenchymal stem cells were discovered by Safwat et al. [67]. These miRNAs may suppress angiogenesis and the inflammatory response in DR.

In addition, it is challenging to guarantee long-term viability and maturity in 3D cultivated organoids because material diffusion and transport are insufficient to fulfill the increasing metabolic requirement. For the proper culture of brain organoids to continue, a functioning circulatory system must be established. Organoid chips can be used to replicate blood arteries with the capacity to perfuse [70–72]. Microfluidic systems that minimize necrotic areas in midbrain organoids by guaranteeing the supply of media through continuous laminar flow [73] a technique that makes use of forced convection and media mixing to enhance nutrient supply have also been developed for brain organoids, as have microchips or micro-bioreactors. Organ chips are therefore a significant way to address the challenges associated with organoid cultivation [68]. Conversely, the microbiome exerts influences on neurodevelopment and the functionality of the central nervous system, which employing co-culture techniques with microbial entities or their by-products within tissue cultures may elucidate these intricate interactions, and the amalgamation of microbiota and immune elements within an organ-on-a-chip platform could also enhance its fidelity as a model [74]. Moreover, the deployment of neural tissue proliferation molecules aligns with the utilization of dorsal forebrain organoids (FGF2) and epidermal growth factor (EGF), as well as ventral forebrain organoids [75]. Upon identification, growth factors were incorporated into the media for both dorsal and ventral forebrain organoids to facilitate neural differentiation and maturation. For purpose of generating oligodendrocytes containing forebrain organoids, additional insulin-like growth factor (IGF), platelet-derived growth factor AA (PDGF-AA), hepatocyte growth factor (HGF) was used during differentiation and maturation. These promising culture conditions and using growth factors will improve the long-term viability and maturation of organoids [76, 77].

The convergence of bioengineering with the organoid domain has facilitated the advancement of automation and miniaturization in detection processes, alongside the capacity for real-time acquisition of biological data during culturing. Bioengineered hydrogels have been developed and evaluated for their efficacy in supporting the three-dimensional cultivation of organoids, albeit they typically exhibit less promotional impact on growth compared to hydrogels derived from the extracellular matrix. A hydrogel constitutes a three-dimensional matrix of insoluble water-containing polymers. The dimensions of the micropores are capable of accurately replicating the size of organoids [78]. Furthermore, the system can be engineered to capture cells or their contents post-experimentation. This facilitates the examination of interactions between various organoids, with the intent to mirror physiological interactions observed in organs.

As to scale up organoid production, automation and miniaturization techniques are born at the right time, expect for the microfluidic systems, recently, an emerging frontier is combining organoids with artificial intelligence (AI) systems, known as “organoid intelligence” [79, 80]. The goal is to use stem cell-derived organoids not only as models, but also as active components integrated into biological hybrid systems that demonstrate cognition and learning. Osteogenesis imperfecta provides an unprecedented opportunity to elucidate neurophysiological mechanisms and advance the pharmacology and toxicology related to the brain. By summarizing the composition of human brain cells [81] and adding structure, OI systems allow direct experimental access to investigate the processes underlying neural signals and network activity. OI also has the potential to inspire new directions in neuromorphic engineering and proceed unprecedented biological computing capabilities [74]. Since the study elucidates how brain organoids exhibit learning and information processing based on dynamic neuronal signals, these findings could guide the development of hardware and scale up organoid production.

The creation and use of brain organoids will remain a crucial topic for the life sciences in the future due to the quick development of new technologies and the increased interest in brain organoids in recent years. These developments have created new opportunities for a better understanding of human brain development, function, evolution, and disease. Although brain organoids can simulate the cellular, molecular and functional characteristics of brain development, due to the challenges associated with maintaining long-term healthy culture, brain organoids created in vitro are mostly limited in their ability to describe embryonic brain properties during their brief culture period. The significant variability in organoid generation and differentiation is a concern. Despite there are a large number of standardizing protocols to improve reproducibility across different laboratories about brain organoids, such as promising culture systems, growth factors and small molecule [75], its research in the field of diabetes remains to be further explored. More research is still needed to build brain organoids with more sophisticated and developed neural networks. Among them, it is anticipated that the creation of functionally vascularized brain organoids will enable the long-term growth of brain organoids. In summary, brain organoids represent a novel technology that has garnered significant attention and quick growth in recent years, presenting both potential and obstacles for research. It is anticipated that as this technology advances, it will offer a crucial resource for comprehending the human brain and examining a wide range of biological and medical issues.

## Data Availability

The datasets used and/or analysed during the current study are available from the corresponding author on reasonable request.
